# Measuring Corrosion on Vehicles, in Real-Time, Using Digital Imaging and Analysis Techniques

**DOI:** 10.3390/ma15093053

**Published:** 2022-04-22

**Authors:** Susan Sawyer-Beaulieu, Edwin Tam, Abdulkadir Hussein

**Affiliations:** 1 Civil and Environmental Engineering, University of Windsor, Windsor, ON N9B 3P4, Canada; edwintam@uwindsor.ca; 2 Department of Mathematics and Statistics, University of Windsor, Windsor, ON N9B 3P4, Canada; ahussein@uwindsor.ca

**Keywords:** vehicle, automobile, corrosion, prevention, measurement, treatment, rust

## Abstract

This research outlines a digital imaging method under development to systemize a rapid in-field corrosion evaluation measure, to evaluate and monitor the degree of corrosion on target corrosion-prone parts on light-duty vehicles. This procedure uses digital imaging to study and compare corrosion levels of 228 vehicles that were treated with aftermarket applications of corrosion prevention products versus 141 vehicles that were untreated. It introduces a Corrosion Index (CI) as a common measure. Single-factor and two-factor analysis of variance (ANOVA) of the digitally-based corrosion measurements show statistically significant correlations between CI and treatment (treated versus untreated), as well as CI, vehicle age, and treatment. The ANOVA results show that the aftermarket-treated vehicles have statistically significantly less corrosion than the untreated vehicles, demonstrating that digital image analysis is a viable method of measuring corrosion on corrosion-prone vehicle parts, offering the potential to monitor and track the performance/efficacy of aftermarket corrosion treatment in real-time.

## 1. Introduction

In 1999, corrosion cost the US transportation industry an estimated USD 29.7 billion annually, with the corrosion of motor vehicles accounting for 79% of this cost or USD 23.4 billion [1]. Corrosion increases the cost of maintaining a vehicle, and can lead to depreciation in value, reduced reliability, safety issues, increased premature repair, and the loss of recoverable and recyclable materials [2]. In North America, corrosion is a major concern for vehicles, especially in the regions of southern Canada and the northern United States, because of the high humidity and the use of roadway de-icing chemicals: both accelerate corrosion significantly [3]. Despite decades of advances in improving corrosion resistance in vehicles, a vibrant industry of aftermarket corrosion protection was developed. At the widespread consumer level, however, there has been no practical development on how to assess the effectiveness of corrosion protection on in-use vehicles that is timely, resource-efficient, and easily implemented by industry participants. As a result, assessing corrosion on vehicles remains largely an ad-hoc exercise based on visual observations about the “rust on a vehicle”. Instead, the research presented herein lays out the important first step in promoting a field-deployable corrosion measurement approach for consumer vehicles.

The findings from an extensive literature survey of previous work in the field are discussed in the following subsections.

### 1.1. Corrosion Prevention and Development

Since the 1970s, advances in coating technologies and materials were introduced by automobile manufacturers to prevent corrosion on vehicles. In addition to corrosion protection, other critical factors driving these advancements include aesthetic characteristics, mass production, cost and environmental requirements, and appearance and durability [4]. Modern automotive coating methods consist of at least five main steps:

1. Pretreatment of the welded sheet metal automotive body structure, i.e., body-in-white (BIW);
2. Electrodeposition (ED) of the anti-corrosion or rust prevention layer on the metal underbody and frames;
3. Sealer application on seams and underbody (i.e., underbody coating or UBC);
4. Primer application;
5. Topcoat applications, i.e., basecoat and clearcoat [4].

Pretreatment removes and cleans excess metals from the body-in-white (BIW) sheet metal surfaces and forms an appropriate surface structure to facilitate the bonding of the electrodeposited ferroalloy zinc-plating corrosion-protection layer [4]. Applying polyvinyl chloride (PVC) or urethane sealers on seams and underbody promotes anti-corrosion, seals water leaks, and minimizes chipping and vibrational noise. Applying surface primers (i.e., water-borne, solvent-borne, or powder) promotes adhesion between the coated surface and basecoat, and improves weather resistance, painted surface appearance, and chipping resistance [4]. The application of the basecoat followed by the clear coat further provides the desired surface properties of color, appearance, gloss, smoothness, and weather resistance [4].

All modern vehicles, when sold, have received anti-corrosion treatment at the manufacturing stage: there are no truly untreated modern vehicles. However, many vehicles exhibit corrosion at some point during their life, depending on conditions of use: the manufacturer-applied protection can degrade if the vehicle is exposed to harsh driving conditions. Therefore, despite the advancements in manufacturing automotive coatings, a variety of aftermarket “anti-rust” products were developed that vehicle owners can choose from and use to help protect their vehicles from corrosion. The variety of aftermarket products includes spray-on rust inhibitors, anti-rust under-coating sealants, spray-on salt removers, and cathodic or anodic protection devices.

Spray-on rust inhibitors typically consist of applying a mineral-based oil that creeps into door seams, folds, joints, and weld spots where corrosion commonly starts. Oil-based sprays displace moisture and can be applied to wet surfaces [5]. This, combined with a thicker gel-type oil for the underbody, wheel wells, and rocker panels, help provide protection against corrosion, including electrical components and brake and fuel lines [5].

Sealants containing tar, wax, or polymers are a “one time” corrosion inhibition application typically applied as a coating on the vehicle underbody [5]. Sealants provide a protective barrier against the water and road salt, but unlike light oil sprays, they do not penetrate as deeply into metal folds, seams, etc., where rust typically begins. Sealant treatments are commonly sold through car dealerships and, to be effective, must be applied when the vehicle is clean and dry [5].

Electronic rust inhibitors (for example, cathodic or anodic protection devices) are another anti-corrosion option typically sold by auto dealers [5]. Although electronic rust inhibitors effectively prevent corrosion of active metals in continuous contact with water, such as on bridges and boats, their effectiveness on vehicles over time has not been proven [5,6].

Fleet vehicle operators often employ aftermarket treatment on the assumption that the added protection can extend the useful service lives of their heavily used vehicles. Moreover, aftermarket corrosion protection can add longevity and resale value and even enhance the recoverability of vehicle parts for salvage by warding off extensive corrosion—a key consideration given the average life of an active vehicle is now just over 12 years [7].

However, evaluating the effectiveness of an anti-corrosion process, and assessing the degree of corrosion on a vehicle, remains an industry-wide challenge. There is no single method for evaluating corrosion on vehicles at the consumer or aftermarket level that has been adopted industry-wide. While there are laboratory-based approaches to study corrosion generation and progression, measuring corrosion of vehicles in practice largely consists of the owner or an automotive technician visually inspecting and then assessing the corrosion, usually on an ad-hoc basis. There is no common, rapid testing that has been adopted widely in the aftermarket segment.

Corrosion testing and measurement are commonly conducted using effective lab-based corrosion testing methods (see Section 1.2) and/or using specialized field measurement techniques, such as radiography, ultrasonic testing, and eddy current, as well as digital image analysis or simple visual observation (see Section 1.3).

### 1.2. Lab-Based Corrosion Testing

A variety of standardized lab-based corrosion testing methods are used to check the corrosion resistance of materials and surface coatings and may be carried out in environmental test chambers. Salt spray and salt fog testing, conducted in environmental test chambers, use high-saline environments to measure the corrosion resistance of products, materials, paints, and coatings over extended periods [6]. Salt spray chambers, in general, are not used to predict the corrosion resistance of a material or coating because it does not replicate real-world corrosive conditions [6,8]. Instead, the salt spray test is used to validate the suitability of metal and/or a coating for corrosion resistance service [6,8]. In addition, it is used in a QA/QC (quality analysis/quality control) role, such as for monitoring the effectiveness of an automotive coating production process (e.g., pretreatment and painting, electroplating, galvanizing, etc.), permitting relatively quick comparisons to be made between actual and expected corrosion resistance [6,8].

Shi et al. [2], for example, tested the performance of anti-corrosion coating products, spray-on corrosion inhibitors, and salt remover products, readily available on the market, to evaluate their performance to minimize the corrosive effects of chloride de-icers on DOT (Department of Transportation) winter application equipment and vehicles. Electrochemical impedance spectroscopy (EIS) and linear polarization (LP) were used to test the performance of each of the products in preventing corrosion of carbon steel test coupons when exposed to MgCl_2_ solutions. In one series of tests, the corrosion protection performance of the products was studied by immersing treated test coupons in MgCl_2_ solutions for two weeks, continuously, followed by EIS measurements. In other tests, product-treated test coupons were cyclically immersed in MgCl_2_ solutions for 40 min, followed by EIS and LP measurements, then air-dried for 22 h, and subsequently power-washed, with this cycle repeated eight times. After several weeks of testing, Shi et al. [2] clearly demonstrated certain aftermarket anti-corrosion products outperformed others.

Xi and Xie [9] conducted several rounds of experiments to evaluate the relative corrosiveness of two commonly used de-icing chemicals, NaCl and MgCl2, on common metal coupons used by the automobile industry, including stainless steel, SS410 and SS304L, aluminum, Al2024 and Al5086, and coated automobile body sheet metal. The first round of tests used the SAE (Society of Automotive Engineers) J2334 test compared to the ASTM B117 test, and the second round compared the SAE J2334 test to the North Pacific States (PNS) modified NACE (National Association of Corrosion Engineers) TM-01-69 test [9].

The SAE J2334 test [10] involved the accelerated cyclical testing of a metal specimen placed in an environmental chamber, exposing the sample to a simulated in-service changing environment, i.e., changing humidity (humid stage), salt application, and evaporation (dry stage). The ASTM B117 test is a continuous salt-spray test performed in an environment-controllable salt spray chamber [11]. The PNS-modified NACE TM-01-69 test was an immersion corrosion testing procedure that simulates the repetitive exposure of metals to chemical de-icers by cyclically immersing metal coupon samples in chemical de-icer solutions for 10 min followed by 50 min exposure in air over a total test period of 72 h [9]. In all three testing methods, corrosion rates were determined, as material loss in mils per year (mpy), according to the following equation, as specified in ASTM G28 [12]:

$$\mathrm{Corrosion}\;\mathrm{Rate},\;\mathrm{mpy}\;=\frac{K\times W}{A\times T\times D},\;$$

where:

K = the constant 3.45 × 10^6^ mils per year (mpy),
T = exposure time, in hours,
A = area in cm^2^,
W = mass loss in grams,
D = density of the material tested in g/cm^2^.

In the various tests conducted, Xi and Xie [9] found conflicting results. In the Phase 1 tests, the SAE J2334 test indicated that MgCl_2_ was more corrosive than NaCl, but the ASTM B117 test showed that MgCl_2_ was less corrosive than NaCl. Similarly, in the Phase 2 tests, the SAE J2334 test indicated MgCl_2_ to be more corrosive than NaCl (consistent with the Phase I testing), but the NACE TM-01-69 test was found MgCl_2_ is less corrosive than NaCl [9]. Additional systematic testing, using adjustments to the experimental test parameters (e.g., change in chemical solution concentration used for SAE J2334; modification of testing temperature, immersion time, testing period for NACE TM-01-69, etc.), showed that the inconsistencies in the test results were not the result of differences in chloride solution concentration, different immersion times, testing periods, or testing temperatures [9]. Instead, the inconsistencies were the result of different moisture conditions and differences in the properties of the two salts under high humidity conditions. Xi and Xie [9] concluded that, depending on the in-service environmental conditions that automotive components are exposed to, MgCl_2_ is more corrosive than NaCl in a humid environment, and NaCl is more corrosive under immersion and in an arid environment.

Lab-based corrosion testing methods are, therefore, useful for evaluating the incidence of corrosion on metals, under simulated varying environmental conditions, with and without the presence of de-icing chemicals. They are also likely more applicable to evaluating specific corrosion prevention technologies on a general performance level. Lab-based corrosion testing methods, however, have limitations; they cannot be used practically to measure corrosion on individual vehicles under real-time field conditions or constraints. The challenge remains: how to develop and deploy a practical field assessment method for vehicle corrosion.

### 1.3. Corrosion Measurement in the Field

For the vast majority of practical situations, vehicle corrosion is commonly evaluated by visual inspection. A technician observes the car and informs the customer which part is rusted and what problems could arise [13]. At a minimum, the vehicle owner might simply observe rust developing. Visual assessment of corrosion, however, is unlikely to be reliable or consistent because of: (1) the lack of measurement and performance standards; (2) questionable accuracy and effectiveness, depending on a technician’s or observer’s experience; and (3) the variability of results as a consequence of different environmental conditions such as illumination [14]. Even the technician’s state of mind could be an influencing factor during the assessment process [13].

There are various materials testing approaches for assessing corrosion that was previously explored. Bardal and Drugli [8] discussed corrosion detection methods, including visual inspection, radiography, ultrasonic testing, and eddy current. Radiography uses short wave electromagnetic beams generated from radioactive isotopes to detect the thickness of the material [8]. Ultrasonic testing is similar to radiography but uses ultrasonic waves (sonic wave frequencies of 1–6 MHz) instead of electromagnetic beams [8]. The eddy current method that is useful for detecting corrosion cracking and pitting processes is based on the generation of electrical eddy currents in the surface of a metallic object placed in a field of an electrical coil fed with an alternating current (typically 10 kHz) [8,15]. When the energized coil is scanned across the material surface, the presence of any flaws (such as corrosion) and changes in the material’s physical properties, geometry, and conductivity, will interrupt or reduce the eddy current flow, causing a reduction in the loading on the coil and increasing its effective impedance. Consequently, by monitoring the voltage across the coil, changes in amplitude and phase shift can be used to show changes in material properties, including the presence of corrosion [8,15].

Bardal and Drugli [8] discussed the suitability of these methods in accessible versus non-accessible areas. Although radiography is suitable for both accessible and non-accessible surfaces, visual inspection is only suitable for accessible surfaces. The ultrasonic and eddy current methods are helpful for inspecting non-accessible areas but are not applicable for accessible surfaces [8]. Given the practical constraints, however, to efficiently and cost-effectively assess corrosion on consumer vehicles, and because corrosion is often first detected visually by the owner or an automotive technician, a method based on photographic inspection likely offers the most advantages [13].

Furthermore, corrosion may not appear to cover a significant area on the surface but instead may penetrate below the surface, reducing the metal thickness and producing a color change and/or higher surface roughness [13]. Determining whether these parameters are suitable corrosion indicators or not requires data that measure such occurrences. Currently, three alternative methods are considered to be effective for measuring corrosion on vehicles: (1) thickness analysis; (2) surface-roughness analysis; and (3) digital image analysis [13]:

1. Thickness analysis

This method focuses on the amount of metal lost due to corrosion. Eddy current instruments were successfully used for detecting the thickness of rust in aluminum alloy skins and adjacent fastener/rivet holes of aircraft [16,17,18]. Corrosion thickness can be measured by this type of instrument because corrosion products are not electrically conductive [19]. Although eddy current instruments are effectively used for detecting rust or cracks in aluminum alloys surfaces, corrosion in steel is not normally detectable with eddy currents [19]. Consequently, eddy current method detection would not be considered suitable for measuring total corrosion on a vehicle (which remains predominantly steel).

2. Surface-roughness analysis

In order to measure the surface roughness of a vehicle part, particularly the bottom of the vehicle, a suitable surface roughness tester is needed that will be portable, is a non-contact, areal-type tester, and can measure a suitable range of roughness [13]. A small portable handheld device is preferred to facilitate measurements on both the vehicle’s sides and underside. The surface of a vehicle may have dust, mud, or grease on it, which can interfere with or damage the tester. Moreover, physical contact with a testing device may, in turn, damage the surface of the vehicle. With an areal-type instrument, the degree of surface roughness is measured over an arbitrary rectangular range, giving a more accurate grasp of the state of the surface [13].

The range of roughness that can be measured by the instrument should be large enough (e.g., almost smooth to very rough) to accommodate the differences in roughness that may be found on a rusted metal surface. Some rusted surfaces may have a large surface roughness that, ironically, can be too rough to be measured by some roughness measurement devices. Depending on the testing device, the surface roughness may only be measured successfully on a metal surface that is slightly rusted. Since the experiment to measure the actual roughness of a rusted metal surface has yet to be undertaken, parameters from other metal surfaces can be used as references. For example, a metal surface prepared by sawing will have an average roughness, Ra, in the range of 1.6 to 25 µm [20].

3. Digital Image Analysis

Digital image analysis was successfully used by prior researchers to study corrosion on different machines and products made of metal. In civil and structural applications, it can detect the rust on steel structures, such as bridges and external and internal steel parts of boat hulls [21,22,23,24]. Electric power companies used digital analysis to assess the reusability of steel utility pole crossarms based on their corroded condition [14]. This method involves using specialized image analysis software to detect and extract information from a digital picture (e.g., texture, color, size), which is then analyzed to isolate the rusted area within the picture. This method is considered a fast, convenient, and objective measure of the rust on surfaces [25]. Khayatazad et al. [24] present probably the most comprehensive approach to date using digital image analysis. Their analysis involves assessing the corrosion on 31 images representing corroded steel surfaces. In their approach, the reference mask had to be manually generated, and it is not fully explained if their procedure is easily adaptable to on-site deployment. Nevertheless, their outcomes strongly support a digital imaging method.

### 1.4. Review Summary and Objectives

Based on the review presented in Section 1.2 and Section 1.3, there are several conclusions and interpretations that can be made of the existing state of corrosion assessment. First, from an operational perspective, any field-based approach for measuring corrosion must be:

- Time-efficient. The technician assessing corrosion should be able to perform the assessment within a matter of minutes;
- Conceptually understandable. The technician should be able to grasp the basic approach of the corrosion technique and, if necessary, explain the fundamental premise to a vehicle owner, who also must, in turn, be able to understand and appreciate what is taking place. From this perspective, while it may be desirable to undertake a detailed corrosion assessment at the surface and below the surface for accuracy and precision, from an aftermarket or consumer perspective, this may not be necessary or even desirable: an approximate but workable measure of corrosion may be sufficient for the typical vehicle owner;
- Operationally functional. The mechanics of facilitating the assessment and the actual physical procedure performed by a technician (or even vehicle owner) must be able to conform to relatively restricted operating space (e.g., under a raised vehicle; unencumbered or untethered device; easy to repeat actions in tight spaces, etc.).

Given the advantages and disadvantages of the current corrosion assessment methods, combined with the need for an on-site assessment, a digital imaging approach is favored. Not only is it important to demonstrate that digital imaging techniques can be used to image, interpret and quantify corrosion in the field quickly, but that the resulting corrosion measurements can be used to monitor corrosion progression in real-time reliably, for example, year-to-year. It is not necessary to monitor corrosion over the entire vehicle. Instead, changes in corrosion can be monitored on specific parts that tend to be the most corrosion-prone, for example, body parts and underbody parts. These essentially serve as a proxy measurement for vehicle corrosion. A reliable, rapid digital-imaging method to quantify vehicle corrosion in the field offers the possibility to study and compare the effectiveness of aftermarket anti-corrosion products in real-time.

Based on the relative merits and challenges of prior methods for vehicles, a digital imaging process could assess vehicle corrosion using the following general steps:

1. Take pictures of the corroded parts and materials using a digital camera or camera-enabled device;
2. Analyze the digital images using software to evaluate the corrosion level by:
  - Detecting the presence of rusted areas in each digital image taken of each vehicle part;
  - Determining the extent of rust and calculating the total rusted area versus non-rusted area within each image.
3. Evaluate the overall corroded condition of the vehicles studied relative to vehicle make, model, model year, history of corrosion treatment, history of vehicle use and care, and so forth.

The overall objective of the research described herein was to develop a preliminary metric and accompanying methodology using a digital imaging and analysis process in the field, to assess the extent of corrosion on a vehicle’s target corrosion-prone parts. This metric can be used to track the effectiveness of aftermarket corrosion prevention and treatment applications [13] and provide vehicle owners with an objective assessment of the corroded condition of their vehicle over time. This research compares corrosion measured on a population of randomly-sampled vehicles that were treated with an aftermarket anti-corrosion product, Krown T40 “Rust Protect”, versus corrosion measured on a population of randomly-sampled, untreated vehicles. The fundamental research demonstrates that digital imaging and measurement of corrosion on corrosion-prone light-duty vehicle parts that received aftermarket corrosion treatment can be used to quantify and identify a statistically significant change in corrosion relative to (1) corrosion treatment (i.e., treated versus untreated) and (2) time (i.e., vehicle age).

The authors’ approach is unique because: (1) a large database of hundreds of images from multiple vehicles and vehicles parts were employed in the research, and (2), rather than focusing on the typical steel “samples” or representative testing plates that were corroded in lab-based tests, the authors’ investigation method focused on corrosion measured on actual vehicle parts. The resulting imagery used in this research reflects the corrosion, the adjacent parts, and the operating environment as a technician would encounter the situation in real-time.

## 2. Materials and Methods

The research involves collecting data on a random sample of both treated and untreated vehicles using digital imaging and customer questionnaires. As previously mentioned, all modern vehicles, when sold, have received anti-corrosion treatment at the manufacturing stage: technically, there are no truly untreated modern vehicles. However, aftermarket corrosion treatment continues to serve a legitimate demand to further the protection of vehicles against corrosion. This research focuses on the aftermarket scenarios: the term “treated” refers exclusively to only aftermarket applications of corrosion prevention products and/or processes on a vehicle, while “untreated” refers to vehicles that have only had the anti-corrosion treatment provided by the manufacturer (Hu, 2016), not applications of aftermarket corrosion-prevention products.

Data were collected during two sampling campaigns (referred to as Phases I and II) between 2014 and 2016. Data from a total of 228 Krown-treated vehicles were collected through the course of the two sampling campaigns (67 during the Phase 1 sampling and another 161 during Phase II). Data from another 141 untreated vehicles were similarly collected (104 during the Phase 1 sampling and another 37 during Phase II); both treated and untreated vehicles varied in make, model, and model year.

The data on the 228 Krown-treated vehicles were collected at three Krown facilities in Ontario. The 141 untreated vehicles that were sampled consisted of high-salvage end-of-life vehicles collected for recycling at Standard Auto Wreckers and A&L Auto Recyclers in Ontario, as well as untreated vehicles brought into the Krown facilities for first-time Krown treatment or other automotive services (e.g., tire rotation; oil change).

With the exception of the end-of-life vehicles sampled at the automotive recycling facilities, vehicle owners were surveyed about the history of each vehicle, including driving practices (e.g., city versus highway), driving conditions (e.g., exposure to salted winter roadways or unpaved/gravel roadways), storage, corrosion protection, maintenance, and repair. These survey activities were reviewed and approved by the University of Windsor’s Research Ethics Board.

The treated vehicles have a history of being previously treated with Krown’s T40 “Rust Protect” solvent-free, oil-based corrosion prevention product. A small number of these Krown-treated vehicles may have also had an alternative aftermarket corrosion protection method used on them (i.e., undercoating and/or cathodic/anodic protection devices).

**Figure 1:**
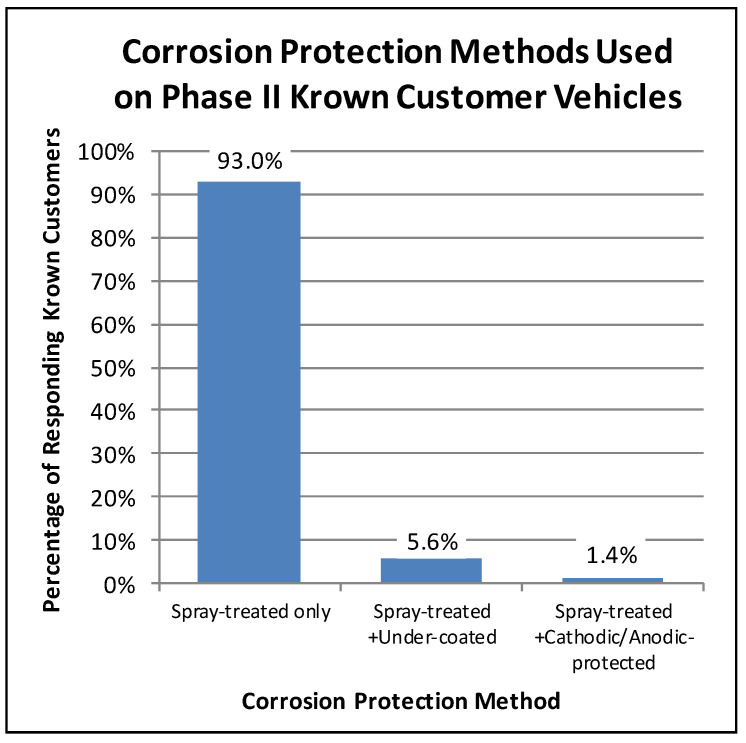
Proportions of Phase II surveyed customers’ vehicles having received different combinations of corrosion treatments.

For example, in the Phase II customer surveys, the Krown customers were asked what corrosion protection methods had been used on their vehicles. Eighty-seven percent of the Phase II participating Krown customers responded with the corrosion protection history of their vehicles. Of these respondents, less than 7% had used an alternative corrosion protection method on their vehicle in addition to the Krown spray treatment. As illustrated in Figure 1, the majority of the responding Krown customers (93%) only had Krown spray treatment. A little less than 6% of the responding Krown customers had their vehicles spray-treated and undercoated, and less than 2% had used a combination of spray-treatment and cathodic/anodic protection. Even though a small fraction of vehicles had received a combination of corrosion treatments, they were still treated. Hence for this research, a treated vehicle was defined as one having been treated with Krown’s T40, with or without the use of an alternative aftermarket corrosion protection method.

**Figure 2:**
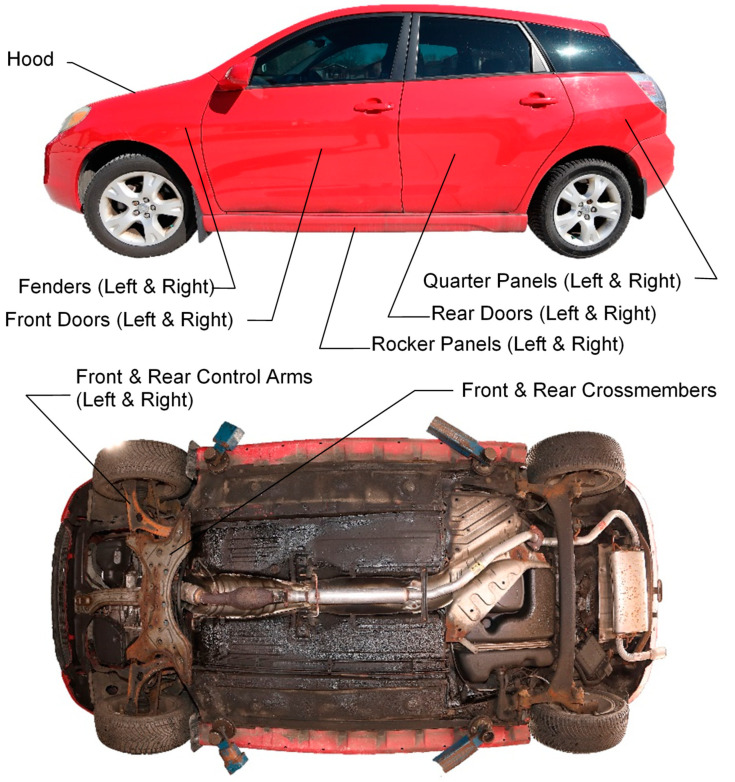
Corrosion-prone body panels and underbody parts used for corrosion study.

Data collection on each vehicle involved inspecting and photographing observed corrosion on 17 targeted part types that are known to typically corrode: 11 body panel part types and 6 underbody part types (refer to Table 1 and Figure 2).

### 2.1. Measurement Process

Digital pictures of the target vehicle parts were taken with either a Nikon Coolpix P700 or a Canon PowerShot G5X camera, and each was equipped with an Aputure Amaran AL-H160 On-Camera LED light assembly. The angle, proper lighting, and clarity (resolution) of the photos taken were key parameters affecting the quality of the data. During the sampling program, sufficient time had to be given to each vehicle (approximately 20–25 min were allotted for analyzing each vehicle), and the lighting was adjusted so that the corroded areas were clearly visible to facilitate an accurate analysis.

**Figure 3:**
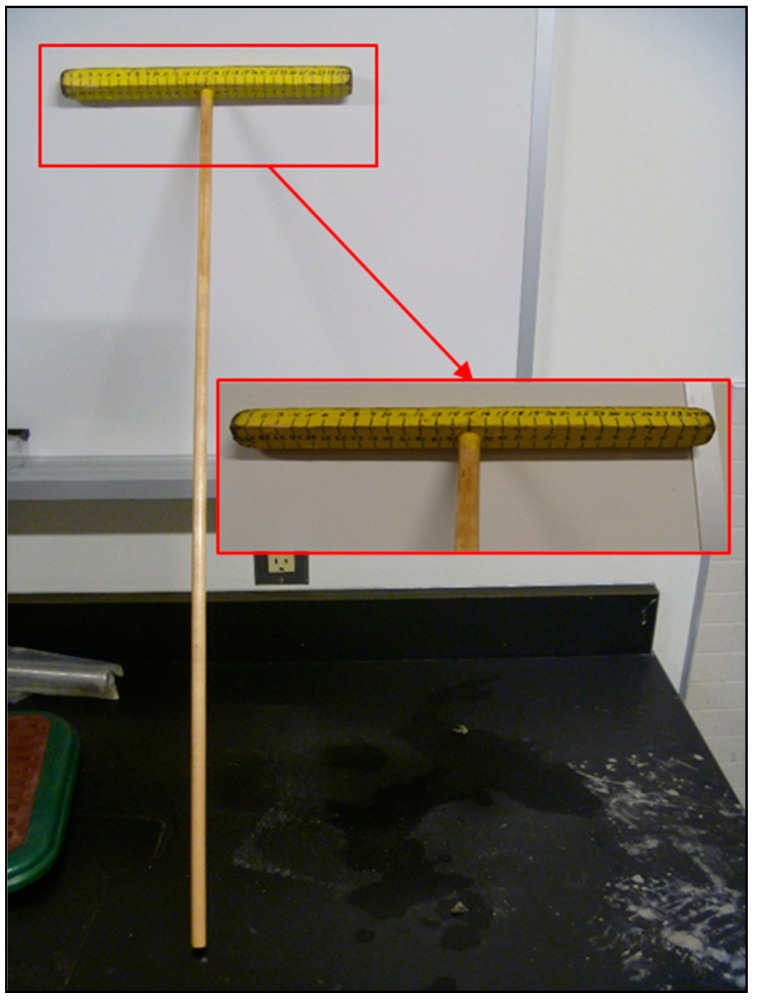
Metric T-scale used as a linear scale when imaging vehicle.

When taking the digital images of the vehicle parts, a metric “T-scale” ruler was used as a reference scale, permitting the measurement of distances and area (see Figure 3). The resulting digital images were then each analyzed using a freeware image analysis software package, AnalyzingDigitalImages (ADI), initially developed by John Pickle and Jacqueline Kirtley (Museum of Science, Boston) and updated by Dan Gullage (STEM Education Institute, University of Massachusetts Amherst) [26].

ADI is part of the image analysis software application, Digital Earth Watch (DEW), which in turn is part of the Global Systems Science curriculum [27], an interdisciplinary, integrated course for high school students emphasizing how scientists work together to understand significant problems of global impact. The DEW software suite provides, for example, digital image analysis tools to analyze Earth images, qualitatively and quantitatively, for changes in ecosystems over time (e.g., deforestation, urban growth, etc.) [27].

**Figure 4:**
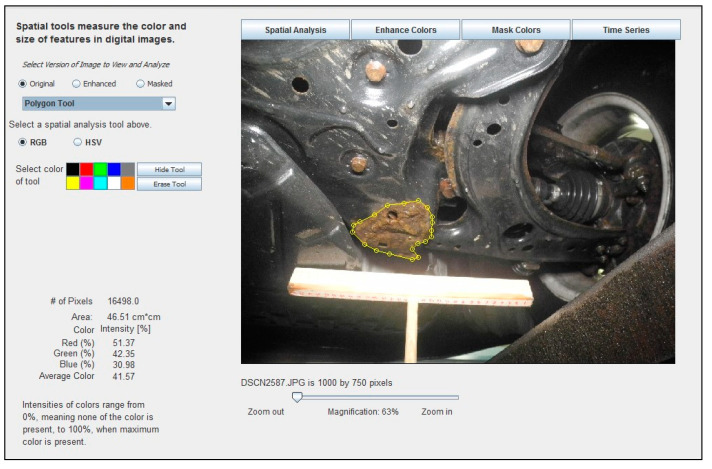
Example of how the ADI software was used to identify, manually delineate, and analyze the corroded area, measuring 46.51 cm^2^, in a digital image of a 2001 Volkswagen Jetta crossmember, measured with the help of a 30 cm long T-scale.

These same software tools can be readily applied to the measurement of corrosion in digital images of vehicle parts. When an image is opened in the software, the image may be trimmed or resized, if necessary, to facilitate faster processing speeds. Image scale may be set using, for example, a known distance represented in the image, areas of corrosion may be outlined using a “Polygon Tool”, and then the corrosion area is automatically calculated. Figure 4 shows an example of how the ADI software was used to identify, manually delineate, and analyze an area of corrosion in a digital image.

**Figure 5:**
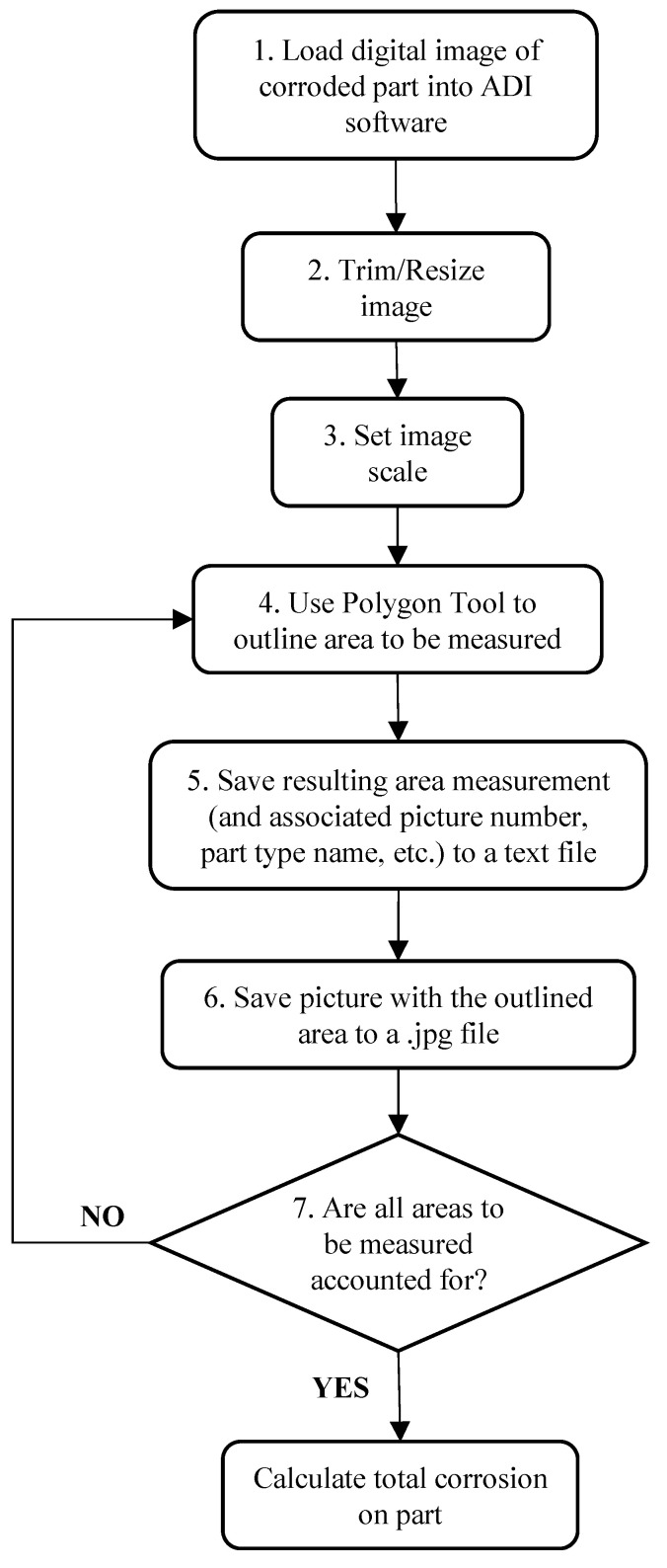
Flowchart of process used to measure corrosion in digital images of vehicle parts, using ADI software.

Areas of corrosion in an image that had to be measured were identified visually, based mainly on color. As illustrated in Figure 5, the measurement process involved (1) loading a photo of a part into the ADI software, (2) trimming or resizing the image if necessary to facilitate faster processing speeds, (3) setting the image scale (i.e., Calibration of Pixel Size), (4) outlining the area to be measured using the Polygon Tool, (5) saving the resulting area measurement (and associated picture number, part type name, etc.) to a text file (for later import into a spreadsheet file for data computation), (6) saving the picture with the area outlined to a.jpg file (as documentation of areas accounted for in CI measurements), and (7) repetition of steps (4)–(6) for each and every area of corrosion that must be accounted for on the particular part in the image. This process was repeated for every photo taken.

For each picture loaded into the ADI software, (1) the pixel length of the 30 cm long T-scale showing in the image was measured, hence setting the image scale in pixels/cm (i.e., Calibration of Pixel Size), (2) each area of visible corrosion was manually outlined using the software’s Polygon Tool, establishing its corroded area in (pixels)^2^, and (3) subsequent conversion and reporting of the area in cm^2^, according to the following equation:

$$\mathrm{Area}\;\mathrm{of}\;\mathrm{Corrosion}\;\mathrm{Measured},{\;\mathrm{cm}}^{2}=\frac{\mathrm{Corroded}\;\mathrm{Area}\;\mathrm{in}\;\mathrm{Image},{\;(\mathrm{pixels})}^{2}}{\mathrm{Area}\;\mathrm{Calibration}\;\mathrm{Factor},{\;(\mathrm{pixels}/\mathrm{cm})}^{2}}$$

### 2.2. Corrosion Assessment

Corrosion on the body panels was identified, measured, and classified according to three different categories of corrosion severity: blistering, surface rust, and perforations [28]. Blistering is considered the mildest form of corrosion because the paint is still present and offers some protection, even though corrosion has started beneath the painted surface. Surface rust is more severe because the protective coatings have essentially failed, and the metal is now exposed and corroding. Perforation is the most severe because the metal has lost part of its integrity and, for the perforated area, no longer offers any protection.

Corrosion on the underbody parts was identified, classified, and measured as surface rust because the underbody parts did not have painted surfaces, and are not subject to blistering, and showed no evidence of perforations [13].

The corrosion measurements are based on the overall Corrosion Index (CI) equation developed by Hu [13]:

(1) CI=(P×Wp)+(B×Wb)+(S×Ws)

where

P = perforation area;
Wp = weighting factor assigned to perforations;
B = blister area;
Wb = weighting factor assigned to blistering;
S = surface rust area;
Ws = weighting factor assigned to surface rust; and
Wp = Wb = Ws = 1.

All areas were measured in cm^2^. The weighting factors represent the severity of each corrosion category compared to one another. For the Phase I and II research, weighting factors of “1” were used, representing the simplest relationship where blistering, surface rust, and perforation are assumed to have equal severity [13]. The authors acknowledge that the use of equal weighting is simplistic: this initial assumption was used given the uncertainty in creating a consumer-appropriate metric that was previously untried. There were also practical limitations in the testing capability. Finally, vehicle owners may pragmatically only be concerned about corrosion when it is visible. Nevertheless, the use of alternate weighting factor values can be explored in future research.

## 3. Results and Analysis

The corroded area measurements determined for each part type for the 369 vehicles sampled were subsequently summarized and analyzed using Excel and Minitab. The corrosion measurements for each vehicle were summed by a group of part types, specifically, (a) Body Panels, (b) Underbody parts, and (c) Underbody + Body Panels (i.e., Total), and expressed as the Corrosion Index (CI) in cm^2^.

### 3.1. Single-Factor ANOVA: Corrosion Index versus Treatment

**Figure 6:**
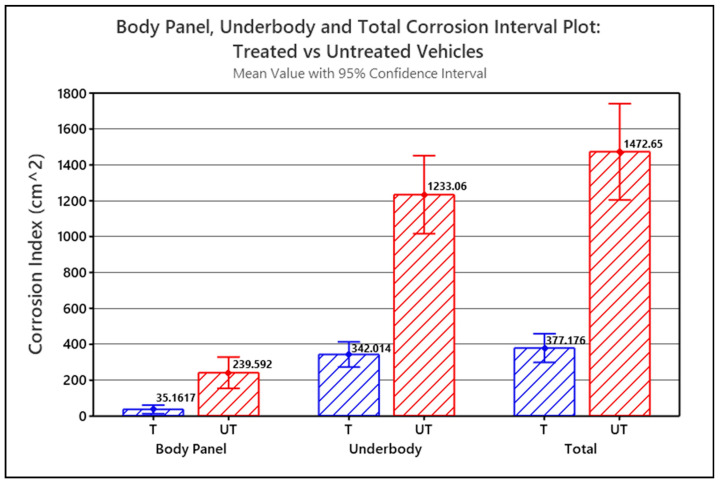
Interval plot for Body Panel corrosion, Underbody corrosion and Total Corrosion (i.e., Underbody + Body Panels) comparing treated (T) versus untreated (UT) vehicles (*p* < 0.001).

The Corrosion Index values for each of the part type groups for the treated and untreated vehicles were statistically analyzed using a single-factor (a.k.a. one-way) analysis of variance (ANOVA) and compared to identify significant correlations between Corrosion Index and whether the vehicle was treated or untreated. ANOVA was used to test if the means of the two groups were significantly different or not. Figure 6 graphically illustrates the single-factor ANOVA results, comparing mean Corrosion Indices versus treatment, calculated for the treated vehicles (T) and untreated vehicles (UT) for each part type group.

Table 2 summarizes the one-way ANOVA analysis statistics for the three scenarios of corrosion index versus treatment. In each one-way ANOVA analysis, the null hypothesis, “that all means are equal”, and the alternative hypothesis, “at least one mean is different”, were tested using a level of significance, α = 0.05. o determine the significance of the null hypothesis, the probability value, *p*, was compared to α:

- If *p* ≤ α, the null hypothesis is rejected in favor of the alternative hypothesis. Meaning the differences between the means are statistically significant;
- If *p* > α, accept the null hypothesis as true, i.e., the differences between the means are not statistically significant.

The Corrosion Index means shown in Figure 6 are all calculated with a 95% confidence. The error bars in the graph represent the standard error of the mean (SEM). In all three scenarios, *p* < 0.001, and since *p* < α in each case, the difference between the means for the treated versus untreated vehicles is statistically significant. In all three scenarios, the graph shows that the corrosion-prone parts of the treated vehicles statistically have significantly less corrosion than the corrosion-prone parts of the untreated vehicles. Based on the corrosion index means, the untreated Body Panels had 6.8 times more measurable corrosion than the treated Body Panels, and the untreated Underbody parts had 3.6 times more measurable corrosion than the treated Underbody parts. The combined untreated Underbody + Body Panels had 3.9 times more corrosion than the treated Underbody + Body Panels.

The underbody corrosion index dominates the combined Underbody + Body Panel corrosion index simply because significantly more corrosion was measured on the Underbody parts than on the Body Panels. The treated Underbody parts had, on average, 9.7 times more corrosion than the treated Body Panels. The untreated Underbody parts had 5.2 times more corrosion, on average than the untreated Body Panels.

### 3.2. Two-Factor ANOVA: Corrosion Index versus Vehicle Age Group and Treatment

**Figure 7:**
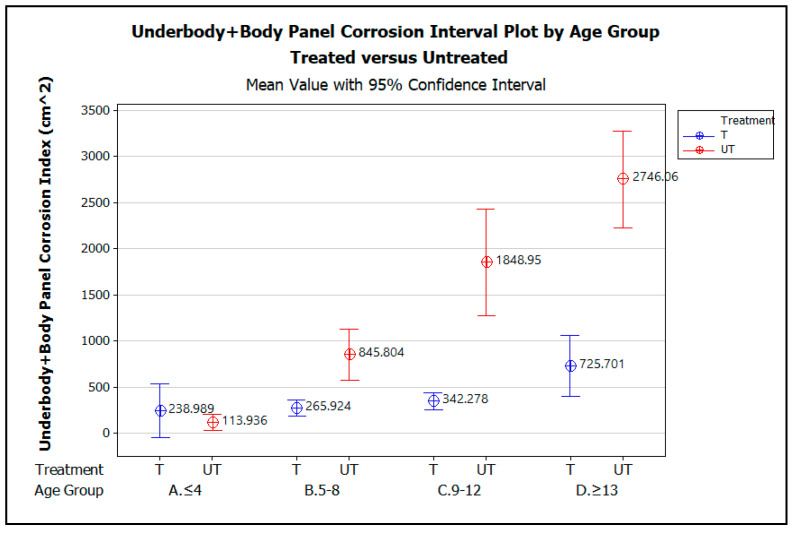
Underbody + Body Panel corrosion interval plot by vehicle age group, A. ≤4 yrs, B. 5–8 yrs, C. 9–12 yrs, and D. ≥13 yrs, comparing treated versus untreated vehicles.

Two-factor (a.k.a. two-way) ANOVA was used to establish if there is an interaction between the two independent variables, vehicle age (by age group) and treatment (i.e., treated and untreated), on the dependent variable, corrosion index. Figure 7 graphically illustrates the comparison of the mean corrosion indices for Underbody + Body Panels of the treated vehicles (T) and untreated vehicles (UT), plotted against the four vehicle age groups, ≤4 yrs, 5–8 yrs, 9–12 yrs, and ≥13 yrs. Table 3 summarizes the two-factor ANOVA analysis statistics for the three scenarios of corrosion index versus treatment. Again, the Corrosion Index means are all calculated with a 95% confidence interval and α = 0.05.

The graph demonstrates that for vehicles up to 4 years of age, there is no statistically significant difference in the amount of corrosion measured on the treated versus untreated vehicles, as indicated by the way the error bars for the treated vehicles overlap those for the untreated vehicle. The lack of a statistically significant difference in measured corrosion on the treated and untreated vehicles within this age group is likely due to the effectiveness of the OEM corrosion prevention measures applied when these early model vehicles were manufactured. From the ages of 5 to 13 years and beyond, however, the graphs show that, statistically, significantly less corrosion was measured on the treated vehicles than the untreated vehicles. Although the amount of corrosion increases with vehicle age for both the treated and untreated vehicles, the increase in corrosion with increasing vehicle age is greater on average for the untreated vehicles than for the treated.

## 4. Discussion

By using analysis of variance (ANOVA), the preliminary statistical analyses of the Corrosion Index (CI) values for the Body Panels, the Underbody parts, and the Underbody + Body Panels for treated and untreated vehicles identified statistically significant correlations between Corrosion Index and treatment (treated versus untreated) as well as between Corrosion index, vehicle age group, and treatment. The ANOVA results demonstrate that the corrosion measurements made using the digital image analysis methodology are statistically significant. Less corrosion was measured on the treated corrosion-prone vehicle parts than on the untreated corrosion-prone vehicle parts, confirming that the digital image analysis process applied in this research is a viable method of measuring corrosion on vehicles. The ability to measure and track the effectiveness of corrosion prevention is critical given the increasing life of active vehicles and the consequences of preventable corrosion. The results presented are directly applicable to assessing actual vehicle components that corrode in the environment.

### Error Sources

During the course of the research, several researchers were tasked with taking the pictures and processing them using the ADI software. Although the different personnel received direct training to consistently identify the parts of interest, use the equipment to take the pictures, and use the software to process the images, there were nevertheless observational errors associated with the predominantly manual interpretation of the digital imaging and analysis. As a result, these operator-associated errors were suspected of contributing to variances in the CI measurements, as suggested by the error bars shown in Figure 5 and Figure 6. Sources of errors include, for example:

- Misidentification of a part; for example, a rear axle being mistaken for a rear crossmember;
- Pictures were taken at angles that deviated from 90° to the observation plane;
- Differences in how the corroded area measurements in the digital images could be undertaken. For example, in one method, corroded areas on a part are measured directly with the ADI software and were then summed. In a second method, corrosion-free areas were measured, summed, and subtracted from the overall part area. The operator’s interpretation of what was corroded or not corroded, as well as how precisely the areas of corrosion in the digital images were manually delineated using the measurement tools in the ADI software, could also differ.
- The use of an alternative aftermarket corrosion protection method, i.e., undercoating and/or cathodic/anodic protection devices, in combination with the Krown’s T40 corrosion treatment, may also introduce a bias in the comparisons between treated and untreated populations. According to the Phase II survey respondents (response rate = 87%), only 7% of the vehicles had received a combination of aftermarket corrosion treatments. To alleviate this source of bias, we defined treated vehicles as all vehicles having been treated with Krown’s T40, with or without the use of an alternative aftermarket corrosion protection method. In the future, such biases may be avoided by obtaining more samples of that group of vehicles and including the prior treatment as a factor in the ANOVA.

As part of ongoing follow-up research, observational error sources are being reviewed, assessed, and corrective steps were identified to improve the measurement accuracy and repeatability. Nevertheless, even with the potential discrepancies in the corrosion measurements made with the mostly manual digital image analysis process, there is a clear statistically-significant trend identified, showing significantly less corrosion observed on treated vehicle parts than on untreated parts.

## 5. Conclusions

The ultimate objective of this research was to incorporate this process into a system that would be readily accessible to automotive technicians in the field to (1) rapidly assess and facilitate monitoring of the corrosion condition of different treated vehicles over time and (2) track the performance/efficacy of aftermarket corrosion treatment.

The following summarizes future follow-up research activities being undertaken:

1. Statistical analyses are being performed to study and identify factors with a significant influence on corrosion of treated versus untreated vehicles, as well as factors that do not. For example, ANOVA and/or correlation analyses will be performed to determine if any of the following factors significantly affect the incidence of corrosion on the targeted corrosion-prone parts of the treated and untreated vehicles:
  - Vehicle age;
  - Driving practices (e.g., city versus highway);
  - Driving conditions (e.g., exposure to salted winter roadways or unpaved/gravel roadways);
  - Storage practices (e.g., in a garage or not);
  - Maintenance practices (e.g., frequency of washing), etc.;
  - Using combinations of alternative aftermarket corrosion protection methods.
2. Corrosion correlation analyses are being performed to determine if specific Body Panel or Underbody parts, or a combination of parts, can serve as a proxy for overall corrosion on a vehicle: in other words, can measuring one select part describe the state of corrosion for the entire vehicle meaningfully, and thus save measuring multiple body parts;
3. Additional analysis techniques, such as the automated interpretation and assessment of color in digital images using a computer-based algorithm, are under development to reduce the reliance on the operator and thus decrease errors introduced by human judgment. While accepted imaging techniques were used, future research will focus on refining the capability to distinguish corrosion better using, for example, improved corrosion color recognition and analysis, as well as the development of a mobile platform for field deployment. Other possible corrosion assessment techniques, such as AI and Machine Learning (ML) approaches, will also be investigated in future research activities;
4. The research provides statistically significant confirmation that the optical method of digitally recording and measuring corrosion on specified corrosion-prone parts of vehicles is feasible and that, in terms of corrosion, untreated vehicles fare worse in general than aftermarket-treated vehicles. Nevertheless, the method that has been developed for measuring vehicle corrosion would benefit from additional studies to mitigate errors. The corrosion measurement method can be refined, and error sources reduced. The resulting refined methodology would be tested and validated by sampling and analyzing the corrosion on another group/population of vehicles, e.g., 160 aftermarket corrosion treated vehicles and 160 untreated vehicles, with 40 vehicles in each of the four age groups, ≤4 yrs, 5–8 yrs, 9–12 yrs and ≥13 yrs. Two-way ANOVA would be used to determine if the refined method gives results with similar CI versus age-group trends and reduced confidence interval error bars.

The anticipated overall objective of future research will be to determine whether changes in corrosion occurrence on a vehicle can be measured and monitored over time using the digital imaging analysis techniques tested in this research. Although all modern vehicles receive anti-corrosion treatment at the manufacturing stage, the effectiveness of the OEM-applied treatments and coatings can deteriorate over time if, for example, the vehicle coatings become chipped, exposing the underlying bare steel to oxygen and humidity. Therefore, aftermarket corrosion prevention products help minimize the development of corrosion and its progression; however, aftermarket solutions often require annual or periodic maintenance and renewal.

The potential to image, assess, and track the progress of ongoing treatment versus the extent of corrosion (e.g., increasing significantly, increasing minimally, no change) can provide insight into the circumstances where substantial aftermarket treatment can make a notable difference in maintaining the safe, operational longevity of the vehicle. Such tracking of corrosion over time would be of practical use to an individual vehicle owner by demonstrating how a straightforward and relatively low-cost option such as aftermarket corrosion treatment can add value and operational robustness to the vehicle. Further, this practical application would be of particular interest to fleet operators in order to better manage and prolong the useful service life of multiple vehicles that may be operating in harsh environments. The facilities referenced in this research often treat fleet vehicles, and the capability to demonstrate the real and measured results of aftermarket corrosion prevention would assist significantly in developing and justifying effective fleet maintenance routines.

## Figures and Tables

**Table 1:** Targeted corrosion-prone part types used for corrosion assessment.

Body Panels:	Underbody:
1. Hood	12. Front Crossmember
2. Right Fender	13. Rear Crossmember
3. Left Fender	14. Left Front Control Arm
4. Left Front Door	15. Right Front Control Arm
5. Right Front Door	16. Left Rear Control Arm
6. Left Rear Door	17. Right Rear Control Arm
7. Right Rear Door	
8. Left Quarter Panel	
9. Right Quarter Panel	
10. Left Rocker Panel	
11. Right Rocker Panel	

**Table 2:** Summary of statistics for single-factor ANOVA analysis of corrosion index versus treatment for treated and untreated vehicles.

ANOVA Scenario	1	2	3
ANOVA Statistics	Total Body Panel Corrosion vs. Treatment	Total Underbody Corrosion vs. Treatment	Total Underbody + Body Panel Corrosion vs. Treatment
Significance level α	0.05	0.05	0.05
F-value	28.87	83.41	85.19
*p*-value	<0.001	<0.001	<0.001
R^2^	0.0729	0.1852	0.1884
Adjusted R^2^	0.0704	0.1830	0.1862
Sample Count—Treated	228	228	228
Sample Count—Untreated	141	141	141
Mean—Treated	35.2	342.0	377.2
Means—Untreated	239.6	1233	1473
Standard Deviation of the Mean—Treated	185.1	535.7	612.8
Standard Deviation of the Mean—Untreated	524.5	1307	1615

**Table 3:** Summary of statistics for two-factor ANOVA analysis of corrosion index versus vehicle age group and treatment for treated and untreated vehicles.

		Underbody + Body Panel Corrosion vs. Treatment	
ANOVA Statistics		Treated	Untreated
Significance level α		0.05	
F-value		18.78	
P-value		<0.001	
R^2^		0.4655	
Adjusted R^2^		0.4551	
Sample Count, by Age Group	A. ≤4 yrs	19	32
	B. 5–8 yrs	81	36
	C. 9–12 yrs	86	30
	D. ≥13 yrs	42	43
Mean, by Age Group	A. ≤4 yrs	238.98	113.93
	B. 5–8 yrs	265.92	845.80
	C. 9–12 yrs	342.28	1848.95
	D. ≥13 yrs	725.70	2746.06

## Data Availability

The data are not publicly available due to the proprietary nature of the vehicle images and the integrally-related vehicle owner’s information and survey data.
